# Evaluation of the cross-protective effect of VR2332 modified live virus vaccine against a recombinant NADC34-like porcine reproductive and respiratory syndrome virus

**DOI:** 10.3389/fvets.2024.1472960

**Published:** 2024-11-20

**Authors:** Yu Wu, Limiao Lin, Xiaopeng Gao, Jiaying Zheng, Lijuan Yin, Haishen Zhao, Bohua Ren, Lianxiang Wang, Qunhui Li

**Affiliations:** ^1^Wen's Food Group, Yunfu, China; ^2^State Key Laboratory of Biocontrol, Guangzhou Higher Education Mega Center, School of Life Sciences, Sun Yat-sen University, Guangzhou, China; ^3^College of Animal Science, Guangdong Provincial Key Lab of Agro-Animal Genomics and Molecular Breeding, South China Agricultural University, Guangzhou, China

**Keywords:** porcine reproductive and respiratory syndrome virus, modified live virus, NADC34-like strain, cross-protection, epidemiology

## Abstract

In recent years, NADC34-like strains of porcine reproductive and respiratory syndrome virus have gradually emerged as mainstream strains on Chinese pig farms. These strains have high mutation rates and can recombine with local strains, representing great challenges to prevention and control efforts. Previously, a new recombinant NADC34-like subtype strain was isolated in our laboratory. Herein, we evaluated the cross-protective effect of the VR2332 modified live virus (MLV) against the novel NADC34-like recombinant strain using the immune challenge protection test in piglets and sows. The results revealed that immunization with the vaccine in piglets significantly reduced viremia, lung damage and stimulated the production of PRRSV-N antibodies. In the sow challenge experiment, one abortion and one death were recorded in the positive control group, and the survival rate of offspring was only 25%. However, there were no sow deaths or abortions in the immunization group during the experiment, and the average piglet survival rate was high at 76.5%. In general, the VR2332 MLV confers a certain extent of cross-protection against the NADC34-like recombinant strain, providing an effective reference and guidance for prevention and control efforts and clinical vaccine use.

## 1 Introduction

Porcine reproductive and respiratory syndrome (PRRS) is an acute infectious disease of pigs caused by PRRS virus (PRRSV) (1). Clinically, PRRSV mainly causes fever, respiratory symptoms, and death in piglets. Most importantly, the virus can cause reproductive disorders such as abortion and stillbirth in sows, leading to huge losses in the pig industry in China and globally (2–4). In 1987, the disease first emerged in the United States; subsequently, European scientists isolated the first strain of PRRSV named Lelystad strain, which is the classic European I strain (5). The VR2332 strain was isolated by American scientists in 1992, after which PRRS spread globally (6, 7). In China, PRRSV was first isolated in 1996, and other PRRSV subtypes have been continuously reported (8, 9). After over 20 years of evolution, the genetic diversity of PRRSV has become extremely complicated (10).

In recent years, the infection rate of NADC34 subtype strains in China has increased annually (11, 12). According to monitoring data, the detection rate of the strain is continuously increasing (13). From 2017 to 2021, the proportion of positive cases increased from 3 to 28.6% (14, 15). This significant upward trend indicates an increase that the prevalence of NADC34 subtype strains in China. Currently, the provinces that have isolated NADC34 strains are mainly concentrated in the northern region, such as Heilongjiang, Henan, Shandong, Hebei, Jilin, Jiangsu, and Liaoning (11, 12, 16–18). However, this does not indicate that the southern provinces are not affected by the strain. Thus, it is necessary to pay close attention to its epidemic trend. NADC34 strains are likely to recombine with local viruses to produce new variants (19). This type of recombinant virus may lead to a wide range of epidemics, posing a more serious threat to China’s pig industry.

To date, vaccination remains a key measure for PRRSV prevention and control. To control the diverse strains of PRRSV, researchers have developed a variety of PRRSV vaccines, which can be divided into three categories: attenuated live vaccines, inactivated vaccines, and genetically engineered vaccines (20). At present, two types of vaccines are mainly used globally against PRRSV: modified live virus vaccines (MLVs) and inactivated vaccines (KVs). However, because of many defects in the conventional vaccine itself coupled with the inherent characteristics of PRRSV, such as its propensity to mutate and antibody-dependent enhancement, there are many problems in the application of conventional PRRSV vaccines, leading to frequent immune failure (21). Therefore, it is extremely important to select a vaccine with broad-spectrum protective effects. It was developed by Boehringer Ingelheim and introduced in China in April 2005 (22). A previous study found that the Ingelvac PRRS MLV has different degrees of protection against different subtypes of strains (23–25).

Therefore, in this study, piglets 3and sows were challenged with a new NADC34-like strain (GD-H1) after immunization with the Ingelvac PRRS MLV, and their clinical symptoms, viral titers, and sow pregnancy outcomes were used to evaluate the protective effects of the vaccine against the new strain, providing a reference and guidance for PRRSV prevention and control efforts.

## 2 Materials and methods

### 2.1 Virus and vaccine

The PRRSV GD-H1 strain was isolated and preserved in our laboratory, and the GenBank reference number is ON691479. The GD-H1 virus was inoculated into Marc-145 cells plated at a density of 90% and cultured in DMEM containing 2% fetal bovine serum. The cytopathic effect was observed. When the extent of cytopathic effect reached 100%, the culture bottle was transferred to a − 80°C freezer. After three freeze–thaw cycles, the virus culture medium was collected and centrifuged at 1000 × *g* for 5 min, and the supernatant was collected. The 50% tissue culture infectious dose was calculated using the Reed and Muench method (1938). The Ingelvac PRRS MLV used in this study was purchased from the Boehringer Ingelheim.

### 2.2 Animal experiments and immune procedures

**Figure 1:**
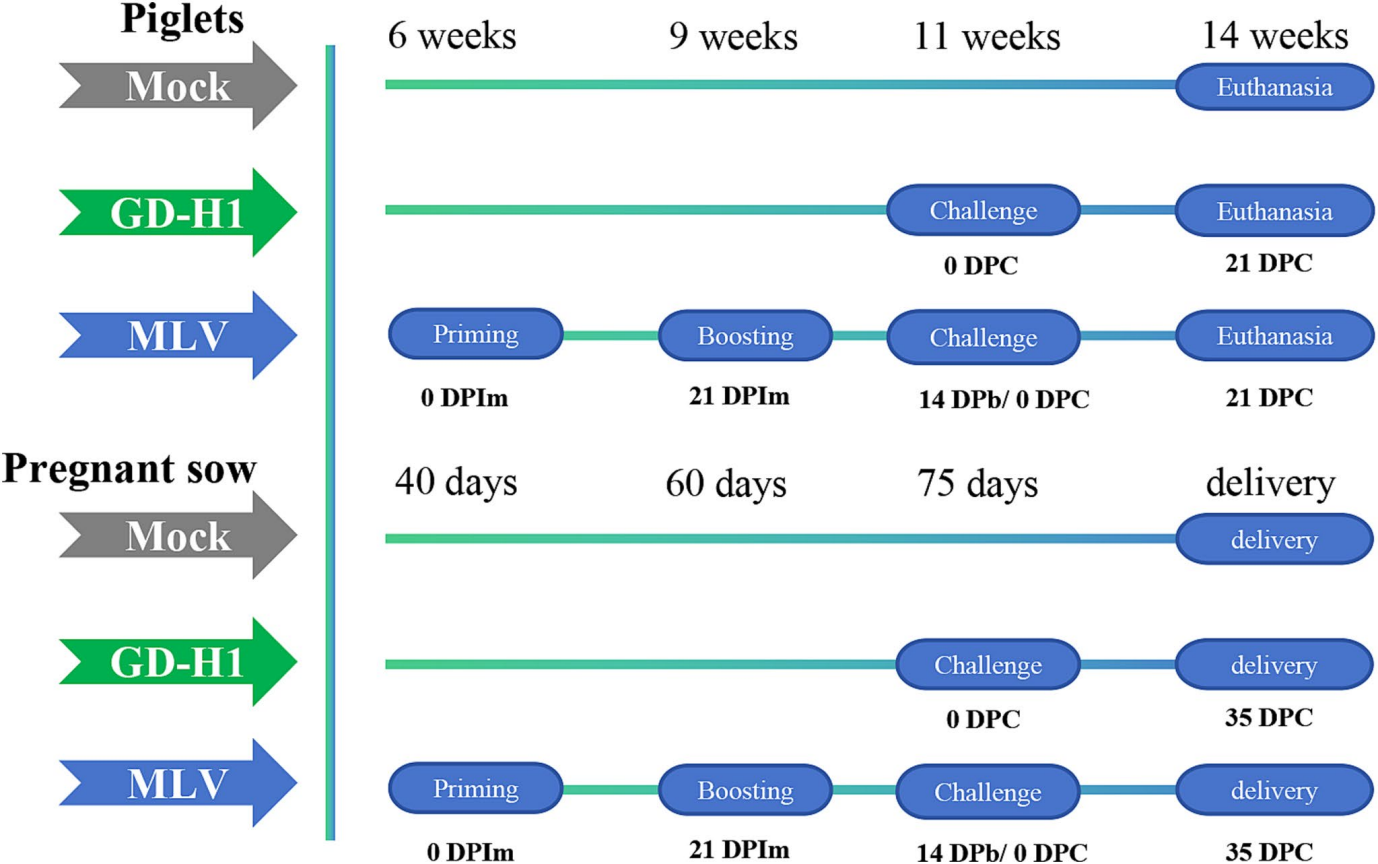
Schematic representation of the experimental design.

In the piglet vaccine evaluation experiment, 15 piglets (6 weeks old) were randomly divided into three groups: blank control group, GD-H1 challenge group, and MLV group (five piglets/group). Piglets in the blank control group did not receive the vaccine or virus. Piglets in the GD-H1 challenge group were challenged once with PRRSV at 11 weeks of age. Piglets in the MLV group were immunized with the vaccine at 6 weeks of age, followed by a second booster dose 3 weeks later, and then after 2 weeks, the piglets were challenged at 11 weeks of age. After infection, the clinical symptoms, body temperature, viremia, and pathological damage of pigs in each group were monitored for 21 days after infection (Figure 1).

In the pregnant sow vaccine evaluation experiment, 12 sows at 40 days of pregnancy were randomly divided into three groups: blank control group, GD-H1 challenge group, and MLV group (four sows/group). Sows in the blank control group did not receive the virus or vaccine. Sows in the GD-H1 group were challenged with the virus at 75 days of gestation. In the MLV group, the sows were immunized with the first vaccine at 40 days of gestation, and they received a booster dose 3 weeks later. Then, the sows were challenged at 75 days of gestation. After infection, clinical symptoms, body temperature, viremia, death rates, and antibody production were monitored in each group. Finally, the delivery of piglets by sows was recorded (Figure 1).

Before the experiment, the piglets and sows used in the experiment were serologically tested using the IDEXX PRRS X3 antibody ELISA kit (IDEXX Laboratory, Westbrook, ME, USA), which confirmed that all animals were negative for PRRSV antibodies. In addition, blood of pigs was screened via reverse transcription–quantitative polymerase chain reaction (RT–qPCR) to clarify the absence of PRRSV and other swine viruses, including swine influenza virus, classical swine fever virus, and transmissible gastroenteritis virus.

### 2.3 Viral RNA extraction from samples

Blood samples were collected from all pigs weekly, and lung tissue samples were collected at 14dpi. RNA was extracted from all samples using the NucleoSpin® Virus RNA extraction kit (Macherey Nagel, Düren, Germany) following the manufacturer’s protocol. Fifty milligrams of each tissue sample were homogenized in 1 mL of TRI Reagent solution (Sigma-Aldrich, St. Louis, MO, USA) using a bead beater (Ezlyzer, Genetix, Mumbai, India) using the NucleoSpin® Virus RNA extraction kit (Macherey Nagel, Düren, Germany) following the manufacturer’s protocol. The elution of RNA was performed in 50 μL of nuclease-free water. The quality and quantity of the extracted RNA were evaluated using a nano spectrophotometer (NanoSpec, VWR, Radnor, PA, USA).

### 2.4 Quantitative detection of the PRRSV genome via RT–qPCR

The quantification of PRRSV viral copy numbers in serum and tissue samples collected at various intervals was performed via one-step RT–qPCR using VeriQuest Probe qPCR Master Mix (Thermo Fisher Scientific, Waltham, MA, USA) in the CFX 96 Realtime PCR detection system C-1000 Touch chassis (Bio-Rad, Hercules, CA, USA) as previously described (8). With the following primers: sense, 5’-CTAGGCCGCAAGTACATYCTG-3′; antisense, 5’-TTCTGCCACCCAACACGA-3′; Aprobe targeting the PRRSV-Ngene (5’-FAM-TGATAACCACGCATTTGTCGTC-CG-BHQ-3) was also employed. The standard curve of the copy number was calculated as follows:


$$\begin{array}{l} \mathrm{CTvalue}=-3.414\times \log_{10}\left({\mathrm{TCID}}_{50}/\mathrm{reaction}\right)\\ \;+36.61;r^{2}\left(\mathrm{correlationcoefficient}\right):1.00 \end{array}$$

### 2.5 Detection of PRRSV specific antibodies in serum

RRSV-specific ELISA antibody titers were measured using Herdcheck PRRSV X3 antibody test (IDEXX Laboratories, Westbrook, ME, USA) as described by the manufacturer.

PRRSV-specific antibody titer was reported as sample-to-positive (S/P) ratios. The serum samples with an S/P ratio of 0.4 or higher were considered positive.

### 2.6 Weight gain detection

The weight of pigs in each group was measured at 0, 7, 14 and 21 days after challenge, and the weight of pigs at 0dpi was used as the base to calculate the weight gain at other time points, and then the percentage of weight gain was calculated. Finally, the average weight gain percentage of pigs in each group was calculated.

### 2.7 Histopathology and lung lesion scoring

At necropsy, the lung tissues were fixed in 10% buffered neutral formalin for hematoxylin and eosin and immunostaining. Staining was automatically performed using a Leica fully automatic staining machine (Leica, Wetzlar, Germany). The sample slides were observed under a microscope at a magnification of 200×. The microscopic lesions present within the lungs at multiple sites were scored as previously described (26). Each lobe is assigned a numerical value that represents the approximate percentage of the volume of the entire lung it occupies. Specifically, 10 points, divided into 5 dorsal and 5 ventral, assigned to the right anterior lobe, right middle lobe, anterior left anterior lobe, and caudal left anterior lobe. In addition, the accessory lobe received 5 points, while the right caudal lobe and the left caudal lobe each scored 27.5 points, with 15 points in the dorsal lobe and 12.5 points in the ventral lobe. This cumulative scoring system adds up to 100 points in total. The gross lung lesion score was estimated and a score reflecting the number of pneumonites in each lobe was given (27).

### 2.8 Statistical analysis

In each experimental group, statistical significance was measured using one-way analysis of variance. Two-sided *p*-values of <0.05 were considered to indicate statistical significance. All point plots were created using GraphPad Prism (v8.0.0) for Windows, GraphPad Software, San Diego, CA, USA, www.graphpad.com, accessed on 23 July 2022.

## 3 Results

### 3.1 Piglet vaccine protection experiment

#### 3.1.1 Clinical symptoms and temperature

**Figure 2:**
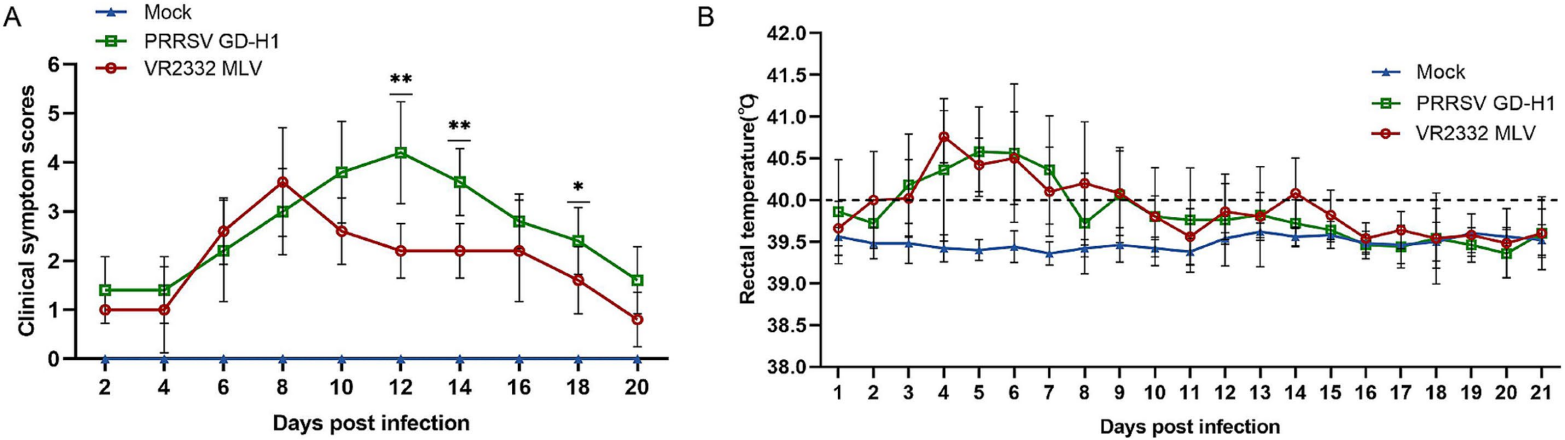
(A) Clinical scores of piglets after challenge throughout the experiment. (B) Body temperature changes in piglets in each group after challenge. Each bar represents the mean ± standard deviation in each group. Significant differences are marked with asterisks: **p < 0.01, and *p < 0.05.

After the end of the immunization program, the piglets were challenged at the age of 11 weeks. After viral challenge, the clinical symptoms and fever of piglets in each group were observed and recorded. The clinical scores were higher in the GD-H1 and MLV groups; however, from the 10th day after the viral challenge, the clinical scores of the MLV group gradually decreased compared with those of the GD-H1 group (Figure 2A). The incidence of clinical symptoms was significantly reduced in the MLV group, indicating that the clinical response of pigs recovered rapidly following immunization. Furthermore, regarding fever, there was no significant difference between the two groups (Figure 2B).

#### 3.1.2 Viremia

**Figure 3:**
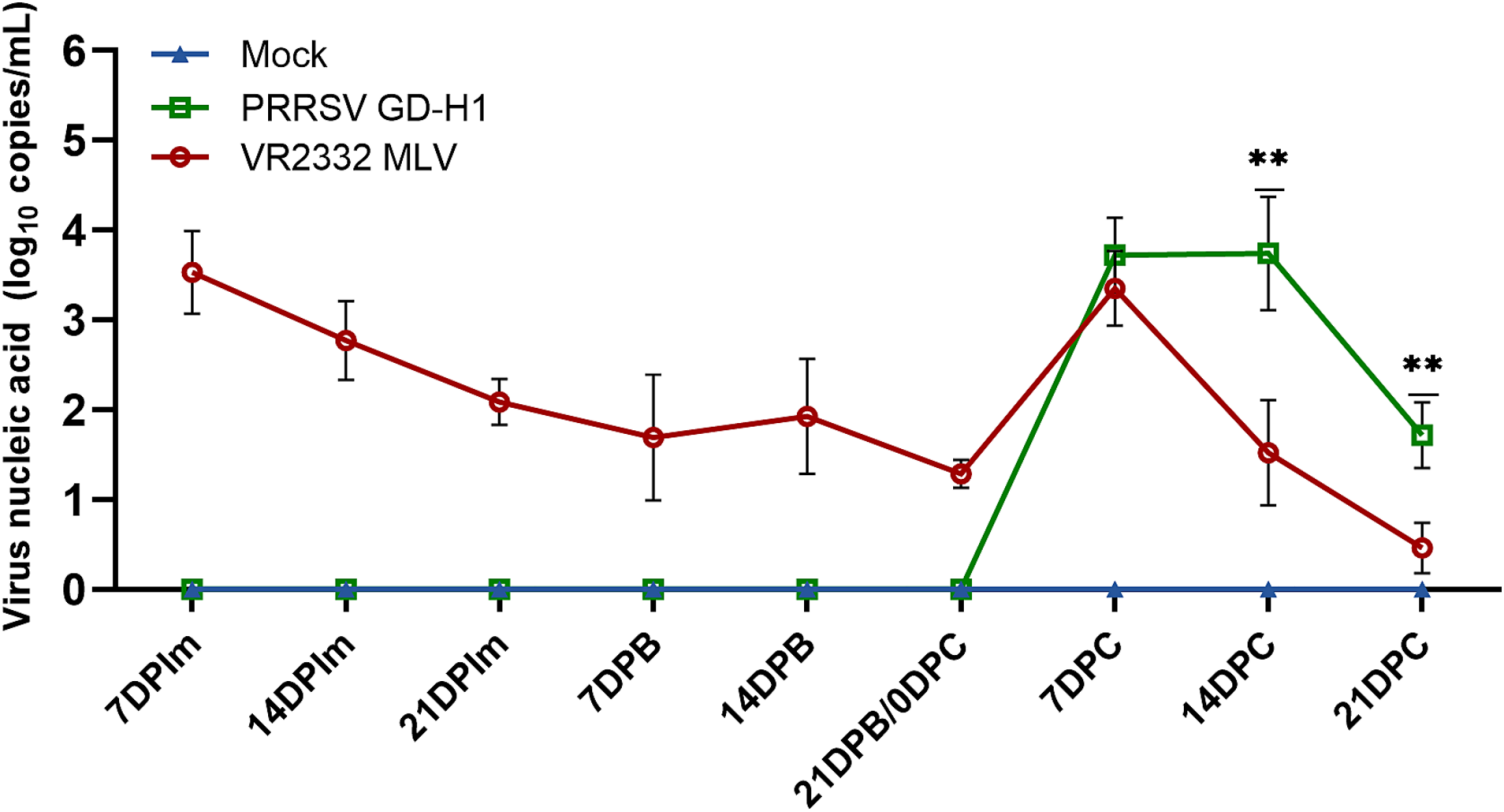
Viral load detection in the blood. Each bar represents the mean ± standard. Deviation in each group. Significant differences are marked with asterisks: **p < 0.01.

The viremia was conducted among the three groups of pigs. The results revealed that the MLV group exhibited viremia after the first vaccine dose, and the level of viremia increased after the challenge. However, the level of viremia after the challenge was higher in the GD-H1 group than in the MLV group (Figure 3).

#### 3.1.3 Observation and detection of pathology at necropsy

**Figure 4:**
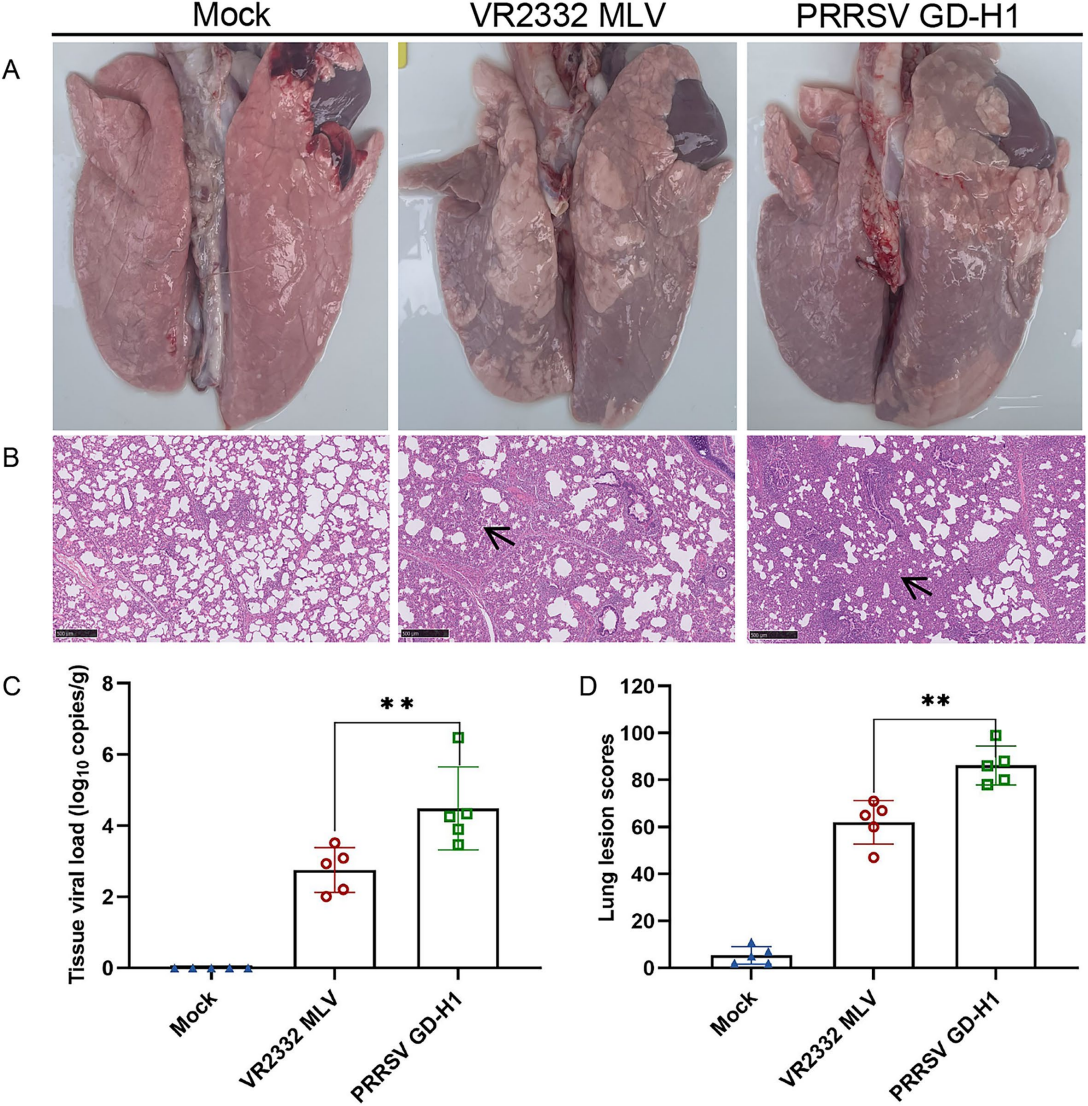
Observation and detection via pathological necropsy. (A) Pathological observation: the lung tissue showed significant consolidation and diffuse hemorrhage after challenged. (B) Histopathology tests: alveolar cell thickened and inflammatory infiltrate. Lesion sites were marked with black arrows. (C) Detection of viral load in lung tissue. (D) Pulmonary Lesion Score.

After viral challenge, the onset of PRRSV is mainly 7 ~ 14 days after infection, the pigs in each group were subjected to lung dissection and pathological examination at 14dpi. The results revealed obvious consolidation in the lungs of pigs in the GD-H1 and MLV groups (Figure 4A). Further histopathological examination illustrated that the alveolar alveolar wall of the infected pigs was significantly thickened, as well as severe inflammatory infiltrates, while no damage was detected in the control pigs (Figure 4B). Meanwhile, the lung score was higher in the GD-H1 group than in the MLV group (Figure 4D). Moreover, the viral load in the lungs was higher in the GD-H1 group than in the MLV group, indicating that vaccination can slow down the infection with the new recombinant strain to a certain extent (Figure 4C).

#### 3.1.4 Anti-PRRSV antibodies

**Figure 5:**
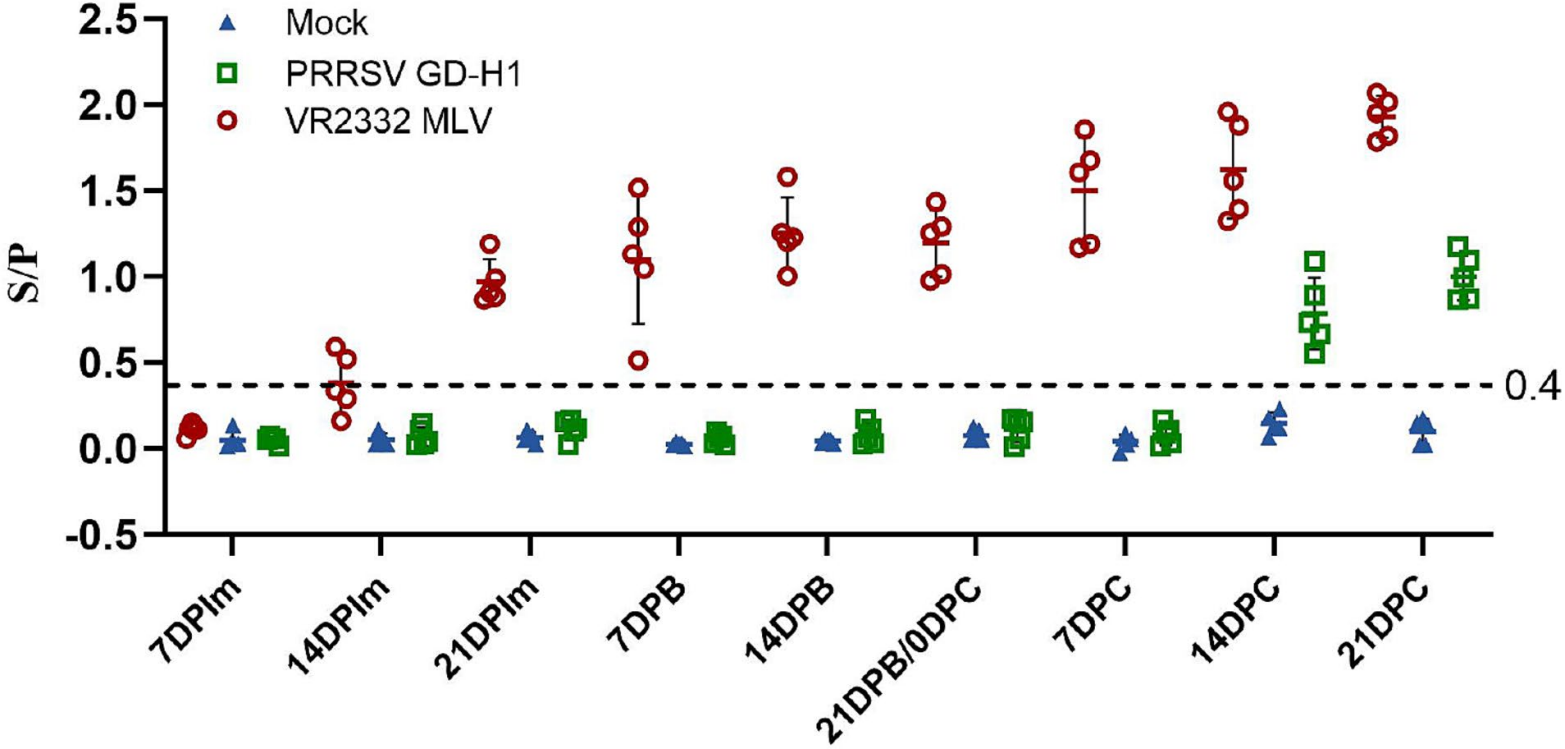
PRRSV-specific antibody level in each group during the entire experment. The serum samples with an S/P ratio of 0.4 or higher were considered positive.

The PRRSV-N protein antibody in the serum of pigs in each group was detected. In the MLV group, PRRSV-N protein antibody began to appear two weeks after the first immunization, and the antibody gradually increased after the second immunization. After the 9th week of challenge, the antibody level of pigs in the MLV group increased significantly. The antibody appeared in the GD-H1 group at the second week after challenge, and was significantly lower than that in the vaccinated group (Figure 5).

#### 3.1.5 Weight gain and survival rate

**Figure 6:**
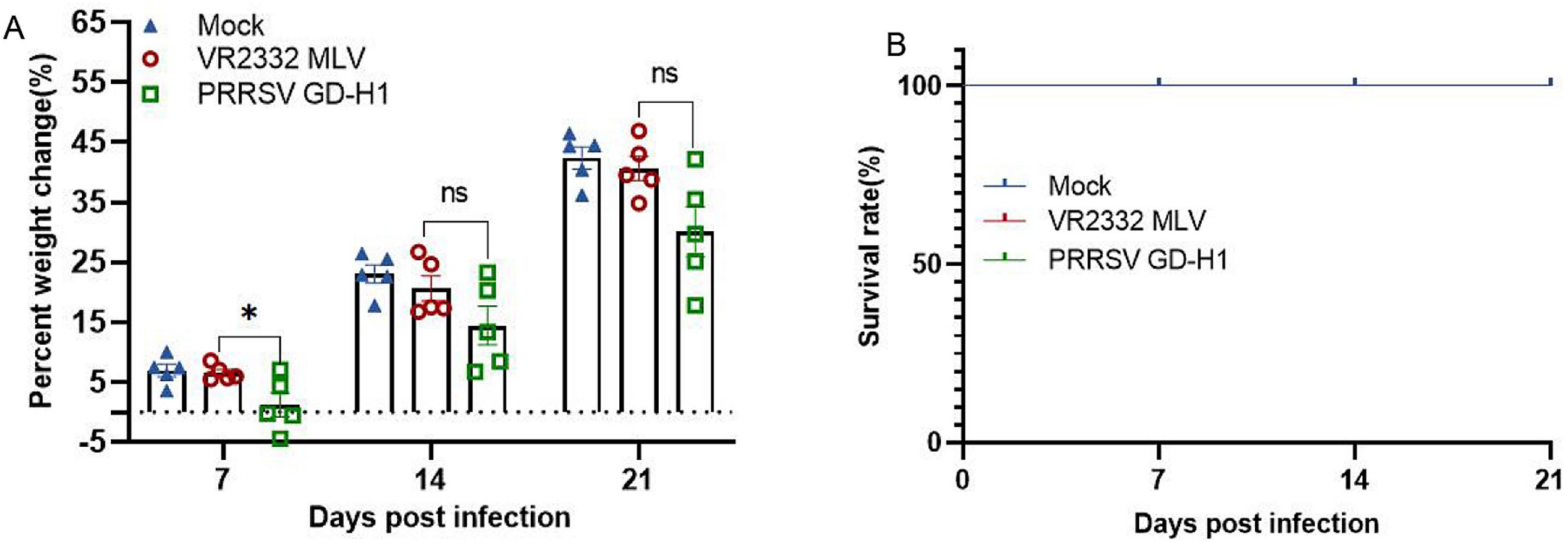
(A) Weight changes of pigs in each group after challenge. (B) Survival rate of pigs in each group during the challenge study. Each bar represents the mean ± standard deviation in each group. Significant differences are marked with asterisks: *p < 0.05.

After viral challenge, the weight and survival of pigs were recorded. Pigs in the MLV group gained significantly higher weight after viral challenge than those in the GD-H1 group at 7dpi. The weight gain was the lowest in the GD-H1 group (Figure 6A). Meanwhile, no pigs died in any group (Figure 6B).

### 3.2 Pregnant sow vaccine evaluation experiment

#### 3.2.1 Temperature and viremia

**Figure 7:**
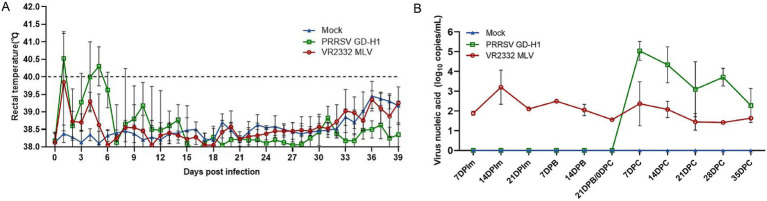
(A) Body temperature changes in pregnant sows in each group after challenge. (B) Viral load detection in the blood. Each bar represents the mean ± standard deviation in each group.

After infection, body temperature was monitored in the sows across all groups. The results indicated that the body temperature of sows in the GD-H1 group rapidly increased after infection but gradually returned to normal on the seventh day. There was no obvious fever in the MLV group (Figure 7A). After viral challenge, viremia was assessed in each group of pigs. Pigs in the MLV group only exhibited viremia after the first dose of the vaccine, and viremia did not increase significantly after viral challenge. Conversely, pigs in the GD-H1 group exhibited higher viremia than those in the MLV group after viral challenge (Figure 7B).

#### 3.2.2 Daily feed intake and antibody

**Figure 8:**
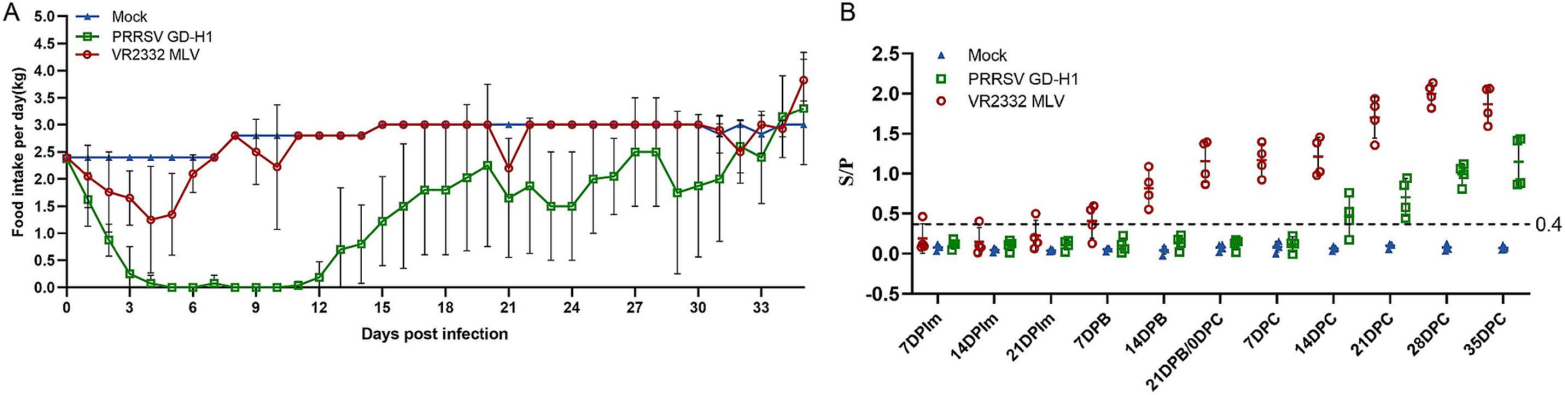
(A) Food intake changes of pregnant sows in each group after challenge. (B) PRRSV-specific antibody level in each group during the challenge study.

The daily feed intake of sows in each group was recorded. The results revealed that the feed intake of sows in the GD-H1 group decreased significantly after viral challenge, and there was little feed intake between days 4 and 12. Subsequently, the feed intake of the animals gradually recovered, but it remained lower than the normal level. The daily feed intake of sows in the MLV group also decreased after viral challenge, but it began to gradually return to normal on the sixth day. No change in feed intake was noted in the blank control group (Figure 8A).

The PRRSV-N antibody was detected in the sera of pigs in each group. The antibody level of sows in the MLV group gradually increased after the second vaccine dose. After viral challenge, the antibody level of pigs in the MLV group increased significantly. The antibody appeared in pigs in the GD-H1 group during the second week after challenge, and but the level was significantly lower than that in the vaccinated group (Figure 8B).

#### 3.2.3 Delivery of sows

After viral challenge, the delivery data of pregnant sows were statistically analyzed. The results revealed one case each of abortion and death among sows in the GD-H1 group. Although the other two sows delivered normally, the survival rate of piglets was only 25%. During the experiment, there were no deaths or abortions in the MLV group, and all four sows completed normal delivery. However, some piglets became ill and died, and the total survival rate was high at 76.5%. The sows in the blank control group delivered normally, and the survival rate of piglets was 90.5% (Table 1).

**Table 1:** Sows delivery data after challenge.

Groups	MOCK	VR2332 MLV	GD-H1
Challenge virus	DMEM	Virus cultures	Virus cultures
Total born	15/11/13/14	15/12/9/11	0/12/11/13
Live born	14/11/11/12	11/9/7/9	0/0/5/4
Stillborn	0/0/0/0	4/3/2/2	0/12/6/9
Mummified	0/0/0/0	0/0/0/0	0/0/0/0
low body weight (< 1 kg)	1/0/2/2	2/3/3/1	−/−/2/3
Piglet survival	90.5%	76.5%	25%
Sow abortions	0/4 (0%)	0/4 (0%)	1/4 (25%)
Sow survival	4/4 (100%)	4/4 (100%)	1/4 (25%)

## 4 Discussion

In recent years, the detection rate of NADC34-like PRRSV has continuously increased in China. Studies have reported that from 2017 to 2019, the detection rate of NADC34-like PRRSV strains was <3%, after which it increased to 11.5% in 2020 and reached 28.6% in 2021 (14, 27). NADC34-like PRRSV has become one of the main epidemic strains in some areas of China along with NADC30-like (35.4%) and HP-PRRSV strains (31.2%) (15). Concurrently, studies have indicated that this subtype strain clinically causes abortion in sows and death among piglets. Specifically, the abortion rate of infected sows is 0–25%, and the mortality rate of infected piglets is 0–80% (28). When a strain recombines with other subtypes, its virulence also changes. A strain isolated in Heilongjiang named PRRSV-ZDXYL-China-2018-2 was associated with a clinical mortality rate of 80% among piglets (29). The difference in pathogenicity between different NADC34 strains might be attributable to the different recombination patterns among the strains (16). In 2021, the TJnh2021 strain was isolated from piglets on a pig farm in Tianjin, China. The results of genetic recombination analysis showed that the strain was a recombinant strain of NADC34-like and QYYZ-like strains. Further animal experiments demonstrated that compared with other NADC34-like strains reported in China, TJnh2021 caused a mortality rate of 40% in piglets and exhibited higher pathogenicity (30). In 2022, a large number of sow abortions and deaths were reported on a pig farm in Guangdong. In our laboratory, a NADC34-like subtype strain was isolated from positive samples. The results of whole-genome sequencing and recombination analysis revealed that the strain was a recombinant strain of NADC34-like and NADC30-like strains. Animal experiments revealed that the strain caused obvious fever in piglets, abortion in 50% of pregnant sows, and deaths in 25% of piglets, leading to serious economic losses to the pig industry (19).

Currently, in addition to strict biosafety measures, vaccination is among the most important means of PRRS control and prevention. The two main vaccine types used globally are MLVs and KVs (31, 32). MLVs have some disadvantages, such as low safety because of continuous replication and shedding in immunized pigs, reversal of virulence, lack of cross-protection against heterologous strains, and weak immune responses (33–35). Compared with live attenuated vaccines, KVs confer less protection and are reported to enhance memory-neutralizing antibody levels and cell-mediated immune responses when exposed to wild-type viruses. In addition, studies have shown that vaccination with MLV results in a strong protective effect against homologous and heterologous PRRSV strains. Among the PRRS MLV vaccines, the 8-subline MLV vaccine is widely used in China. Studies have demonstrated that Ingelvac PRRS and JXA1-R MLVs confer limited cross-protection against NADC30-like strains (36, 37). The 8.7 MLV sublineage vaccines TJM-F92 and R98 confer partial protection against infection with the recombinant NADC30-like FJ1402 strain (38). However, with the emergence of new PRRSV recombinant strains, it is necessary to evaluate whether the existing vaccines confer effective cross-protection against new recombinant strains (37). Therefore, in this study, the most widely used Boehringer MLV vaccine was selected to assess its cross-protection against the new recombinant strain GD-H1 isolated in our laboratory.

In piglets, immunization can significantly reduce viremia and lung damage. In general, for piglets, immunization can reduce the clinical incidence of infection, shorten the course of disease, and reduce viremia. Sow vaccine protection experiments demonstrated that immunization can reduce the incidence of fever and viremia among pregnant sows, significantly resist the decrease in feed intake caused by GD-H1 strain infection, and promote the production of higher antibody levels. The current results supported the efficacy of the vaccine in preventing death and abortion among sows and death and illness among piglets. In general, VR2332 MLV-based immunization can effectively protect pregnant sows from the decline in reproductive capacity caused by infection by new recombinant PRRSV strains and can reduce the risks of death and abortion among sows and the delivery of invalid piglets. This illustrates that the Boehringer MLV has a certain degree of protectiveness against the new NADC34-like recombination strain, consistent with previous studies.

## 5 Conclusion

In this study, the cross-protective effect of VR2332 MLV against a new NADC34-like recombinant PRRSV strain was evaluated using the immune challenge protection test in piglets and sows. The results showed that piglets immunized with the vaccine had reduced viremia, lung damage and produced higher levels of PRRSV-N antibodies. Moreover, sows immunized with VR2332 MLV showed low mortality and abortion rates and reduced delivery of ineffective piglets, indicating that the vaccine can protect pregnant sows from the decline in reproductive capacity caused by infection with the new recombinant PRRSV. In general, the Boehringer vaccine exerted a cross-protective effect against the NADC34-like recombinant strain, and these findings could provide a reference and guidance for prevention and control efforts and clinical vaccine use.

## Data Availability

The datasets presented in this study can be found in online repositories. The names of the repository/repositories and accession number(s) can be found in the article/supplementary material.
